# Outcomes of hip arthroscopy for femoroacetabular impingement: The effect of morphological type and chondrolabral damage

**DOI:** 10.1051/sicotj/2019012

**Published:** 2019-05-22

**Authors:** Hatem Galal Said, Mohammad A. Masoud, Mohamed Mohamed Abdel-Hamid Morsi, Maher A. El-Assal

**Affiliations:** 1 Orthopedic & Trauma Dept. Faculty of Medicine, Assiut University Assiut 71515 Egypt

**Keywords:** FAI, Femoroacetabular impingement, Hip arthroscopy outcomes, Cam impingement, Chondrolabral damage

## Abstract

*Introduction*: Hip arthroscopy for treatment of femoroacetabular impingement (FAI) has shown significant pain and functional improvement. However, the differential outcome of each of the FAI morphological types in contrast to one another remains largely unknown. This study was conducted to detect a possible difference in outcome among different FAI types treated using hip arthroscopy.

*Methods*: In this prospective non randomized comparative study, after exclusion of non-FAI cases and cases with advanced arthritic changes, 90 hips in 85 patients that had hip arthroscopy for the treatment of FAI between 2011 and 2015 in our center were analyzed. The collected patient reported outcome measures (PROMs) included visual analog scale (VAS) of pain, the modified Harris hip score (mHHS), and the non arthritic hip score (NAHS) both preoperatively and at final follow-up. Patient satisfaction was collected at final follow-up. Postoperative PROMs were subjected to three main comparisons based on each of FAI type, labral procedure, and extent of cartilage damage. Repeat comparison based on FAI type after matching of exact chondrolabral condition was also attempted.

*Results*: Mean follow-up was 32.8 months (five patients lost from follow-up). There was a significant improvement in the overall PROMs. This improvement was significantly higher in the cam group in contrast to the mixed group. After matching for chondrolabral condition, this difference was consistent and more evident.

*Discussion*: The outcome of arthroscopic treatment of pure cam FAI is significantly better than that of mixed FAI. Matching of the same chondrolabral condition and repeating the comparison yields similar results.

## Introduction

Femoroacetabular impingement (FAI), a relatively recent clinical entity, has been presented as the cause for hip pain and idiopathic osteoarthritis in non-dysplastic hips [1]. It describes abnormal contact between the acetabular rim and the femoral head neck junction as a result of deformity of the latter rendering the head less spherical (cam impingement) or overhanging acetabular rim (pincer impingement). Pincer impingement is either focal (cranial retroversion) or global (deep acetabulum). In most cases, cam and pincer coexist comprising what is called the mixed FAI [1]. These morphological aberrations can be secondary to childhood conditions such as slipped capital femoral epiphysis or Perthe’s disease. In cases where there is no such detectable condition, high activity and extremes of the range of motion during adolescence could be responsible for the subtle morphological abnormalities leading to FAI later in life [2].

The pathomechanics are variable and depend on the predominant mechanical abnormalities. In cam impingement, the bony bump pushes the acetabular labrum sideways overstuffing the peripheral joint space with a shearing effect on the chondro-labral junction. This leads to earlier chondro-labral junction pathology in cam impingement. In pincer forms, there is earlier labral damage through the mechanical squeezing effect. Cartilage damage is characteristically in the posteromedial part of the joint, usually on the acetabular side (contercoup lesion) [1].

The treatment is primarily operative. It is based on correction of the mechanical error and management of collateral chondro-labral damage. Cam impingement is treated by resection osteoplasty with offset correction of the femoral head neck junction. Pincer impingement is treated in most cases with acetabular rim trimming. In case of labral tear or following surgical detachment or destabilization of the labrum through rim trimming, the labrum is repaired using suture anchors. Labral debridement should be limited to irreparable degenerative or mostly ossified acetabular labrum [3].

According to its location and extent, various methods have been used to address cartilage lesions ranging from simple debridement [4], gluing of cartilage flap [5], microfracture, autologous chondrocyte transplantation [4], osteochondral autograft or allograft [6]. Hip replacement should be, however, considered in patients with marked cartilage damage and advanced degenerative changes.

Hip arthroscopy represents the new gold standard and the least invasive method for management of FAI, yet is the most technically demanding with a steep learning curve. Multiple outcome studies after arthroscopic treatment have been published with follow-ups up to 10 years [7]. Many studies have included the three different FAI types without attempt at differentiation in outcome [8]. Fewer articles have reported outcomes of treatment of single FAI type; pure cam, pure pincer, or mixed FAI only [9, 10]. Direct outcome comparison of the different FAI types is largely missing. This study was conducted in order to detect a possible outcome difference among the different FAI types bearing in mind the collateral chondro-labral damage as a confounding factor.

## Material and methods

This is a retrospective study of a prospectively collected data of our hip arthroscopy series. We reviewed 137 hip arthroscopies that were performed between 2011 and 2015, at our center. Ninety-eight of them were for treatment of FAI. Eight hips were excluded from our study. Exclusion criteria were a history of hip trauma such as a fracture or dislocation, associated hip dysplasia, Perthe’s disease, or advanced arthritic changes (Tönnis Grade 3). This left 90 hips in 85 patients. The data included age, sex, diagnosis, detailed intra-operative notes, pre- and postoperative frog leg view-Alpha angle, and patient reported outcome measures (PROMs) [11]. The collected PROMs included VAS of pain, the mHHS and the NAHS both preoperatively and at final follow-up [12, 13]. Patient satisfaction as a percentage was collected at the final follow-up [14].

An informed written consent was obtained from all patients allowing publication of the collected Data. The approval of the local ethics committee to conduct the study was obtained.

## Hip arthroscopy technique

**Figure 1:**
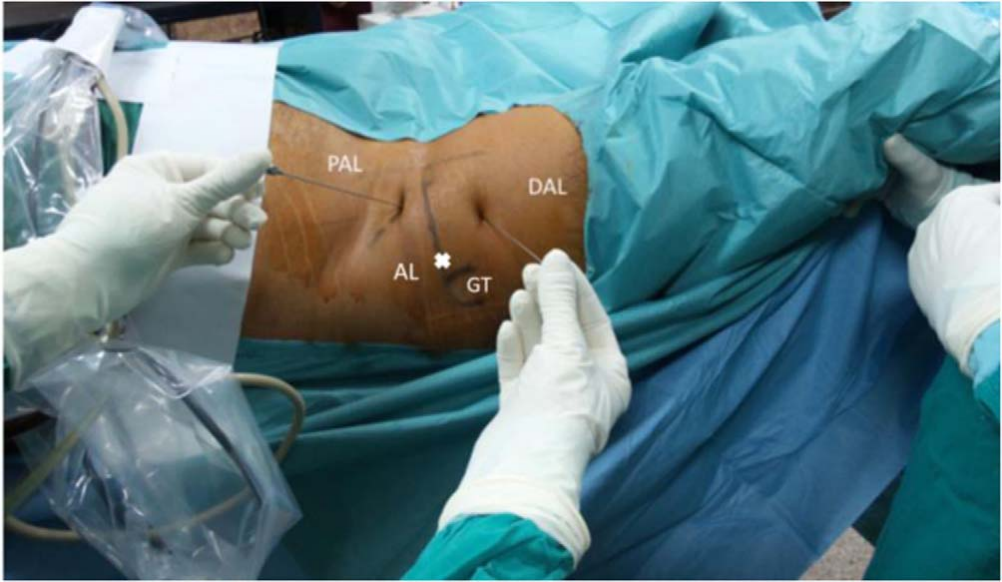
Standard portals used during the arthroscopic procedure. PAL: proximal anterolateral portal, DAL: distal anterolateral portal, AL: anterolateral portal, GT: greater trochanter.

All patients were operated on by the first author. Hip arthroscopy was performed using a standard technique in supine position on a traction table. A peripheral first access is used. The distal anterolateral portal (DAL) is the first portal to be performed. This is followed by the proximal anterolateral portal (PAL). The DAL is made using a triangle method, where the tip of trochanter represents the apex of an isosceles triangle, the other two angles of which are made by the PAL portal and the DAL portal. The PAL portal is then performed at the junction of proximal 1/3 and distal 2/3 of the line between anterior superior iliac spine and greater trochanter tip piercing the capsule under direct vision (Figure 1).

**Figure 2:**
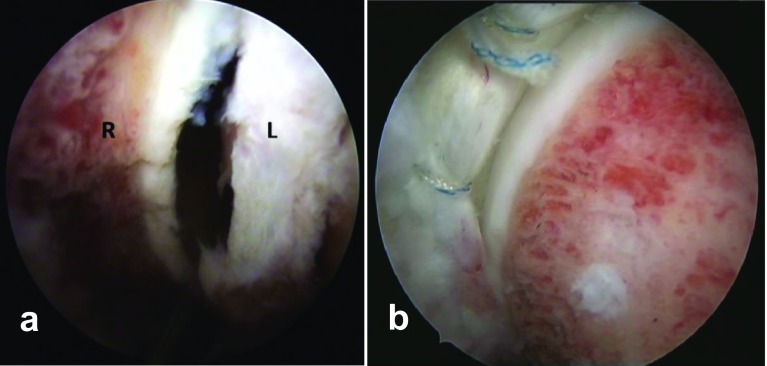
Acetabular rim trimming after labral separation (a). End result after cam osteoplasty and labral repair using suture anchors (b). L: Labrum, R: acetabular rim.

**Figure 3:**
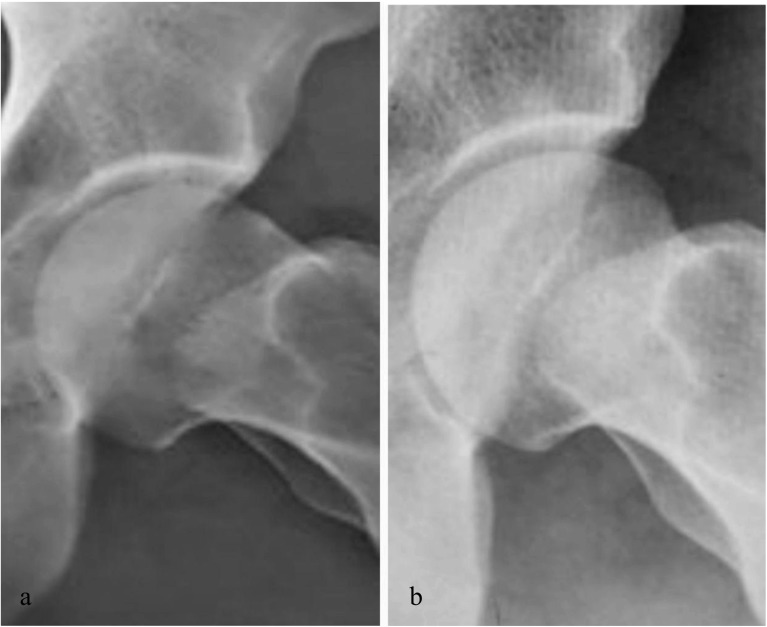
Plain *X*-ray frog view of the left hip joint preoperative (a), postoperative (b) after arthroscopic Cam resection.

The next step is adequate exposure of the anterolateral head neck junction. This is achieved through partial resection of the zona orbicularis and opening of the anterolateral and lateral perilabral sulcus with the hip in 40–50° of flexion. A limited capsulotomy and incision of the iliofemoral ligament is performed with arthroscopy knife or the shaver [15]. The capsulotomy was not repaired in any patient. Cam excision is performed anteriorly and anterolaterally without traction; however, for lateral and dorsolateral cam, traction and internal rotation are required. An alternation of the burr and the scope between the PAL and DAL portal may be necessary. Acetabular edge recession is performed also in the peripheral compartment without traction with the hip in higher degrees of flexion (60–80°) using the DAL portal as a viewing portal and the PAL portal as a working portal. The classic anterolateral portal is then created and traction is applied to allow working more laterally on the rim. Through the anterolateral portal, the insertion of the switching stick under direct vision into the central compartment allows a safe central compartment access; anchor placement for labral refixation can then be performed (Figures 2 and 3). Cartilage reparative procedures were then performed as indicated including either debridement using the motorized shaver or the radiofrequency probe or microfracture in grade 4 lesions with exposed subchondral bone. Postoperatively, weight bearing as tolerated was encouraged in patients that had neither labral repair nor micro-fracture. Twenty KG partial weight bearing was prescribed for 4 weeks in the case of labral repair and non-weight bearing for 6 weeks in the case of micro-fracture for acetabular cartilage defects. Active and passive ranges of motion exercises outside the painful range were encouraged in all patients as soon as possible. Non-steroidal anti-inflammatory drugs were prescribed for 2 weeks postoperative as an ossification prophylaxis.

## Statistical analysis

**Figure 4:**
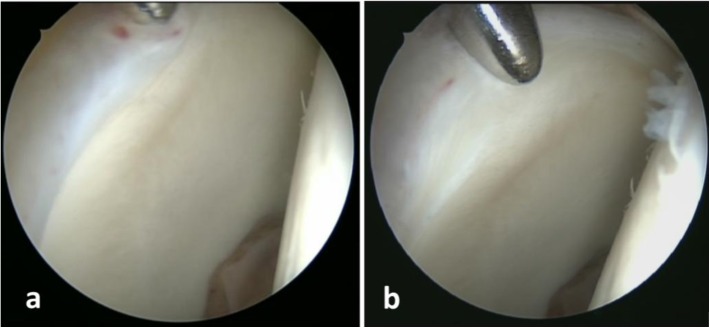
Central compartment of right hip viewed from the anterolateral portal, switching stick is introduced through the anterior portal (a). Cartilage debonding as the stick is pushed against the chondrolabral junction with lesion extending between 1 and 3 O’clock (group 1 cartilage damage) (b).

All statistical tests were performed using IBM SPSS Advanced Statistics 20.0. The Wilcoxon Signed Rank test was used to compare perioperative PROMs. Patients were then allocated to multiple groups in order to compare results for statistical significance. Three main comparisons were undertaken. The first was among mixed, cam, and pincer FAI groups. The second was based on labral intervention. The third was among different cartilage lesion groups. The cartilage damage was grouped according to its size based on the clock face description of the surgeon into three groups, where group 0 had no damage, group 1 had damage that was smaller than 2 h, and group 2 had damage of two or more hours (Figure 4) [16]. Additional subgrouping of different FAI types by matching the specific chondrolabral condition for less confounding result was also attempted. Due to limited sample size, only comparisons with statistical power of ≥ 0.8 were performed. Mann–Whitney test for non-parametric unpaired data was used for the aforementioned comparisons. Statistical significance was considered if *P* ≤ 0.05.

## Results

There were 72 males and 13 females with 46 right, 34 left hips and five bilateral cases. The age ranged from 15 to 67 years (mean: 30.3, standard deviation: 10.3). The mean follow-up was 32.8 months (range 24–72 months, SD = 9.8). Five patients (five hips) were lost from follow-up. The total number in each of the three FAI types and the associated chondro-labral lesions in the remaining 85 hips are shown in Table 1. Table 2 lists the varying morphology of cartilage lesions encountered and the surgical intervention performed. All chondrolabral lesions were located in the ventrolateral quadrant of acetabulum, between 11:00 and 3:00 o’clock. Tables 3 and 4 describe the localization of labral and cartilage lesions, respectively.

**Table 1:** Number of hips in each FAI type and in each subgroup according to chondral and labral lesions.

		Total	Mixed	Cam	Pincer
		85	39	36	10
Cartilage lesion	Type 0	50 (58.8%)	20	22	8
	Type 1	14 (16.5%)	8	6	0
	Type 2	21 (24.7%)	11	8	2
Labral tear	Present	57 (67.1%)	30	22	5
	Absent	28 (32.9%)	9	14	5

**Table 2:** Morphology of cartilage lesions encountered and the surgical intervention performed.

Morphology of cartilage lesion	Number of cases (total 35/85)	Intervention
Wave sign, no chondrolabral separation	16	Radiofrequency stabilization
Chondrolabral separation, no obvious delamination	15	Debridement + Radiofrequency stabilization
Cartilage flap with exposed bone	4	Microfracture

**Table 3:** Location and extent of Labral tears encountered on an imaginary clock face.

Tear extent		No. of hips (57)	%
From	To		
12:00	2:00	14	24.6
1:00	2:00	12	21.1
12:00	3:00	9	15.8
1:00	3:00	4	7
12:00	2:30	3	5.3
2:00	2:30	3	5.3
1:00	1:30	2	3.5
2:00	3:00	2	3.5
12:00	1:00	2	3.5
11:30	2:00	1	<2
11:00	2:00	1	<2
11:00	3:00	1	<2
11:00	12:00	1	<2
10:00	2:00	1	<2
2:30	3:00	1	<2

**Table 4:** Location and extent of cartilage lesions encountered on an imaginary clock face

Lesion size class	Lesion extent		No. of hips (total 35)	%
	From	To		
I (<2 h)	12:00	12:30	1	<3
	1:00	2:00	4	11.4
	12:00	1:00	4	11.4
	2:00	3:00	3	8.6
	1:00	2:30	2	5.7
II (≥2 h)	12:00	2:00	3	8.6
	1:00	3:00	5	14.3
	12:00	2:30	3	8.6
	11:30	2:00	1	<3
	11:00	2:00	2	5.7
	12:00	3:00	5	14.3
	11:30	3:00	1	<3
	11:00	3:00	1	<3

The mean preoperative and postoperative alpha angle, final follow-up PROMs and patient satisfaction are listed in Table 5. The changes in PROMs in the whole series and in each FAI type are listed in Table 6. There was significant improvement of all PROMs in all three types of FAI. The overall patient satisfaction was excellent (>75%) in 52.9% and good (>50%–75%) in 25.9%. In 15.3% it was fair and in 5.9% it was poor (<25%).

**Table 5:** Overall pre- and postoperative Alpha angle and final follow-up PROMs.

	Preoperative*	Postoperative*	*p* value
Alpha angle	69.3 ± 11.9	48.9 ± 11.8	0.001
mHHS	52.1 ± 14.9	71.7 ± 17.4	0.001
NAHS	50.3 ± 16.5	71.2 ± 18.9	0.001
VAS	6.7 ± 1.7	3.1 ± 2.3	0.001
Percent satisfaction	N/A	71.7 ± 21.8%	

* Mean ± SD.

**Table 6:** The change in PROMs (overall and in each FAI type).

	Overall*	Cam*	Mixed*	Pincer*
mHHS	19.5 ± 15.9	25.3 ± 14.3	16.9 ± 13.9	13.5 ± 21.4
NAHS	20.9 ± 19.9	26.7 ± 20.5	18.1 ± 16.6	15.5 ± 25.5
VAS	−3.5 ± 2.6	−4.4 ± 2.0	−2.9 ± 2.6	−3.2 ± 3.5

* Mean ± SD.

Comparing PROMs among different FAI types, the cam group showed statistically significant better results in contrast to the mixed group in each of patient satisfaction (mean ± SD: 76.9 ± 18.5 and 66.9 ± 21.8 respectively, *p* = 0.032), postoperative VAS (mean ± SD: 2.5 ± 2 and 3.6 ± 2.2, respectively, *p* = 0.026), VAS improvement (*p* = 0.015) as well as mHHS improvement (*p* = 0.034).

By comparing the perioperative PROMs and the percent satisfaction among the three groups of cartilage lesion, a statistically significant worse patient satisfaction (*p* = 0.03) was present only in group 2 (>2 h lesions) (mean ± SD: 66.2 ± 21.2) when compared to group 1 (<2 h lesions) (mean ± SD: 80.36 ± 12.63). There was no significant difference in the results among the different cartilage intervention groups (morphologically based).

Labral repair was performed in 38 hips (44.7%), labral debridement in 28 hips (32.9%), and no labral intervention was performed in the remaining cases. There was no statistically significant difference among the three labral intervention groups.

The outcome comparison based on the FAI type after matching once for the exact cartilage lesion group and another time for exact labral condition was performed between cam and mixed groups with intact cartilage, then between cam and mixed groups with torn labrum. Due to limited sample sizes in other subgroups (Table 1) with a statistical power of less than 0.8, no further matched comparisons were performed. For the same reason, a simultaneous matching of the same cartilage and labral condition was not possible.

**Figure 5:**
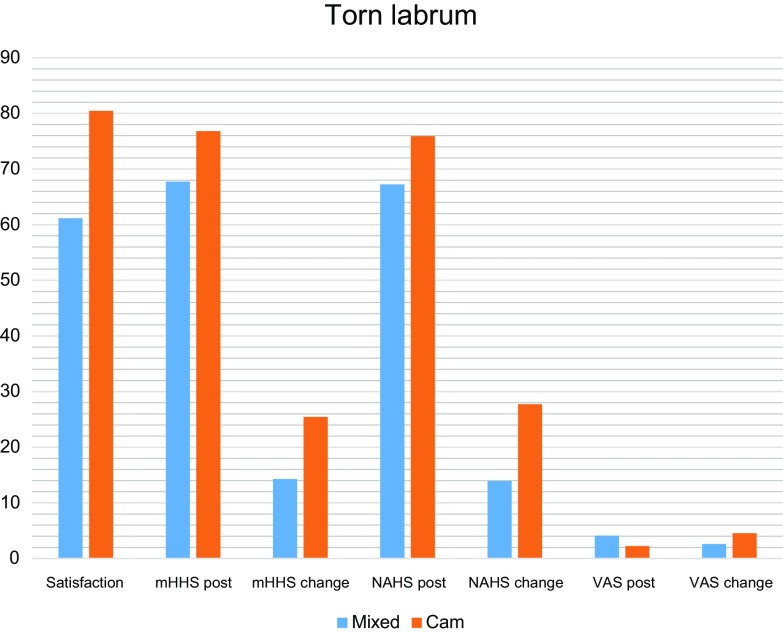
Significant differences found when patients with Cam and torn labrum were compared with those with mixed FAI and torn labrum.

The improvement remained statistically significant for the cam group with intact cartilage in contrast to the mixed group with intact cartilage for both satisfaction (mean ± SD: 78.6 ± 16.7 and 64.8 ± 25.4, respectively, *p* = 0.042), and VAS change (mean ± SD: 4.5 ± 1.6 and 2.5 ± 1.7, respectively, *p* = 0.033). Furthermore, the cam group with labral tear, in contrast to the mixed FAI group with labral tear had a significantly better satisfaction (mean ± SD: 80.5 ± 11.8 and 61.2 ± 20.6, respectively, *p* = 0.001), postoperative VAS (mean ± SD: 2.2 ± 1.5 and 4.1 ± 2.2, respectively, *p* = 0.001), change in VAS (mean ± SD: 4.6 ± 1.3 and 2.5 ± 2.9, respectively, *p* = 0.012), postoperative mHHS (mean ± SD: 76.8 ± 11.7 and 67.7 ± 16.9, respectively, *p* = 0.043), improvement in mHHS (mean ± SD: 25.5 ± 12 and 14.3 ± 14.2, respectively, *p* = 0.008), postoperative NAHS (mean ± SD: 75.9 ± 16.9 and 67.3 ± 15.8, respectively, *p* = 0.047) and the improvement in NAHS (mean ± SD: 27.7 ± 18.2 and 13.9 ± 14, respectively, *p* = 0.005) (Figure 5).

Complications were present in 13 cases (15.3%). Two cases had asymptomatic heterotopic ossification. Nerve palsies comprised the rest of complications. Those included LCFN (six cases), pudendal (two cases), saphenous (one case), and femoral nerve (two cases) palsies. All nerve palsies have started to recover within 6 weeks postoperatively with a complete improvement within 3–4 months except for three LCFN cases that had persistent hypoesthesia in the nerve distribution.

## Discussion

Arthroscopic FAI surgery has resulted in significant pain relief, improvement in PROMs, function, and patient satisfaction [7]. The current study shows an overall improvement in PROMs and satisfaction for FAI patients as a whole. Outcome comparison based on the type of FAI have shown a significantly better patient satisfaction, lower postoperative VAS values, higher mHHS, and VAS improvement of isolated cam patients compared to those with mixed pathology. This difference in outcome could be attributed to a possible higher extent of collateral chondrolabral damage in mixed FAI cases as compared to pure cam cases. By comparing the two types of FAI while matching the same chondrolabral damage extent, the cam outcome remained superior to mixed FAI outcome. In the two matched comparisons that were possible, namely the normal cartilage group in the first comparison and the torn labrum group in the second, this difference in outcome was consistent and more evident in even other outcome measures. Literature search did not yield similar results, no studies that compared outcomes based on the type of FAI were able to be found. Öhlin et al. have investigated a possible correlation between the FAI type (Cam vs. Mixed) and postoperative ihot12 outcome. They could not find a significant correlation but could not exclude type 2 error when rejecting such correlation [17]. However, as a complete matching was difficult and impractical due to small sample size, the collateral damage effect on the outcome difference could not be excluded.

In their survival analysis, Menge et al. have shown the association of acetabular cartilage damage requiring microfracture with reduced survival of the hip joint after arthroscopy [7]. Fontana et al. have reported that the worst results were recorded in cases with a chondral defect equal to or greater than 3 cm^2^ [4]. Larson and Griveans found that all hips that underwent subsequent THA (three cases) had Outerbridge grade IV cartilage delamination greater than 2 cm at the time of FAI surgery [18]. Our findings which show a worse outcome with cartilage lesion exceeding 2 h size on the clock face representation of acetabulum, are in harmony with the above findings. The clock face sizing of cartilage delamination, though subjective, was carried out by the same surgeon who operated all cases.

Previous studies have shown better outcome with labral repair over labral debridement [19]. In the recent work of Menge et al. with 10 years follow-up, they could not detect significant difference between outcomes of labral debridement and labral repair. When they matched for microfracture, though, they found a higher association of labral debridement with poor joint survival. Both labral treatments resulted in significant improvement in those patients who did not require hip arthroplasty [7]. In our work we could not detect significant difference between outcomes of both labral procedures. This could be due to limited sample size.

The overall complication rate in hip arthroscopy reported in the literature ranged from 1.34% to 15% [20]. Traction complications are characteristic to hip arthroscopy and can vary much in severity [21]. Harris reported a major complication rate of 0.58% and a minor complication rate of 7.5% [22]. All complications in this series were minor. Seijas et al. reported a complication rate of 14.34% (37 cases) in their 258 patient cohort [23]. Sharfman et al. have also reported even a higher complication rate (29% in FAI patients) after their use of a special patient questionnaire that dealt in detail with the possible traction complications [24]. In the multicenter prospective study of Larson et al., the overall complication rate has shown to be 8.1% after exclusion of 14.9% that had transient LCFN palsy considering it as a sequel rather than a complication [25].

This study shows that hip arthroscopy for treatment of FAI produced significant pain relief and functional improvement. The improvement of patients with pure cam FAI was significantly better compared to mixed FAI. The confounding effect of collateral chondrolabral damage is a known inherent problem in the FAI outcome research. A further analysis is though needed, ideally with a bigger sample size for better matching of chondrolabral condition.
